# Influence of type 2 diabetes on local production of inflammatory molecules in adults with and without chronic periodontitis: a cross-sectional study

**DOI:** 10.1186/s12903-015-0073-z

**Published:** 2015-07-27

**Authors:** Hasaan G. Mohamed, Shaza B. Idris, Mutaz F. Ahmed, Anne N. Åstrøm, Kamal Mustafa, Salah O. Ibrahim, Manal Mustafa

**Affiliations:** Department of Clinical Dentistry, Faculty of Medicine and Dentistry, University of Bergen, Årstadveien 19, 5009 Bergen, Norway; Department of Oral Rehabilitation, Faculty of Dentistry, University of Khartoum, Khartoum, Sudan; Hamad Medical Corporation, Doha, Qatar; Oral Health Competence Center in Western Norway, Hordaland, Bergen Norway

**Keywords:** Diabetes mellitus, Chronic periodontitis, Inflammation, Gingival crevicular fluid, Cytokines

## Abstract

**Background:**

Pathological changes in periodontal tissues are mediated by the interaction between microorganisms and the host immune-inflammatory response. Hyperglycemia may interfere with this process. The aim of this study was to compare the levels of 27 inflammatory molecules in the gingival crevicular fluid (GCF) of patients with type 2 diabetes, with and without chronic periodontitis, and of chronic periodontitis subjects without diabetes. A putative correlation between glycated haemoglobin (HbA1c) and levels of the inflammatory molecules was also investigated.

**Methods:**

The study population comprised a total of 108 individuals, stratified into: 54 with type 2 diabetes and chronic periodontitis (DM + CP), 30 with chronic periodontitis (CP) and 24 with type 2 diabetes (DM). Participants were interviewed with the aid of structured questionnaire. Periodontal parameters (dental plaque, bleeding on probing and periodontal pocket depth) were recorded. The GCF levels of the 27 inflammatory molecules were measured using multiplex micro-bead immunoassay. A glycated haemoglobin (HbA1c) test was performed for patients with diabetes by boronate affinity chromatography.

**Results:**

After adjustment for potential confounders, the DM + CP group had higher levels of IL-8 and MIP-1β, and lower levels of TNF-α, IL-4, INF-γ, RANTES and IL-7 compared to the CP group. Moreover, the DM + CP group had lower levels of IL-6, IL-7 and G-CSF compared to the DM group. The DM group had higher levels of IL-10, VEGF, and G-CSF compared to the CP group. The levels of MIP-1α and FGF were lower in diabetes patients (regardless of their periodontal status) than in chronic periodontitis subjects without diabetes. Diabetes patients (DM + CP and DM) had higher Th-2/Th-1 ratio compared to the CP group. HbA1c correlated positively with the pro-inflammatory cytokines (Pearson correlation coefficient = 0.27, P value: 0.02).

**Conclusion:**

Type 2 diabetes and chronic periodontitis may influence the GCF levels of inflammatory molecules synergistically as well as independently. Type 2 diabetes was associated with high Th-2/Th-1 ratio, and modulated the local expression of molecules involved in the anti-inflammatory and healing processes.

## Background

Diabetes mellitus represents a heterogeneous group of metabolic disorders in which elevated blood glucose levels result in disturbance of carbohydrate, fat and protein metabolism [1]. The most common form is type 2 diabetes [2]. Diabetes is a major public health concern with 380 million people suffering from the disease worldwide, and about 80 % of the patients are from low- and middle-income countries [3]. It is expected that Africa will take the lead in terms of the largest proportional increase in adults with diabetes by 2030 [4]. Prevalence of diabetes in The Sudan, as in many other low-income countries, is increasing to epidemic proportions [5]. In 2014, the prevalence of the disease in The Sudan was about 18 % [3], which ranks The Sudan among countries with high prevalence of diabetes in Africa and the world. It also reflects the change in life style and the urbanization movement of the population.

Chronic hyperglycemia is associated with irreversible complications such as nephropathy, retinopathy, neuropathy, cardiovascular diseases, peripheral vascular diseases, delayed healing and periodontal diseases [6]. Periodontal diseases, including the reversible form (gingivitis), are highly prevalent and affect up to 90 % of adults worldwide [7]. Chronic periodontitis is characterized by apical migration of the epithelial attachment accompanied by loss of connective tissue and alveolar bone [8]. These changes are mediated by the interaction between pathogens and the host immune-inflammatory response [9]. Although periodontal pathogens are considered as the initiative factor of the disease, [10] tissue destruction in chronic periodontitis is the consequence of the host response to those pathogens [11].

The exact mechanism by which diabetes affects periodontal tissues is not fully elucidated [12]. An altered immune-inflammatory response to bacterial pathogens has been suggested [11]. Hyperglycemia can affect periodontal tissues by increasing oxidative stress as a result of the imbalance between reactive oxygen species and antioxidants, which may eventually lead to accumulation of Advanced Glycation End products (AGE) [13]. The binding of AGE to their receptors (RAGE) triggers intra-cellular events that enhance the production of pro-inflammatory cytokines, chemokines and cell adhesion molecules [14]. Hyperglycemia can be assessed by measuring the concentration of glycated haemoglobin (HbA1c), which reflects mean glucose levels over the previous 8–12 weeks [15].

One means of investigating the local inflammatory status of the oral cavity is by analysis of gingival crevicular fluid (GCF), a non-invasive approach for assessing the presence or absence of various inflammatory molecules [16]. GCF is a transudate or an inflammatory exudate that can be collected from the gingival crevice surrounding the teeth. It contains components of circulating blood, local tissues and most importantly, host-derived inflammatory molecules [16, 17].

Most of the studies of GCF inflammatory molecules in individuals with diabetes have generally been based on small subject samples and investigation of a limited number of inflammatory molecules, with inconclusive results [12]. Multiplex analysis of inflammatory molecules, whereby a large number of inflammatory molecules can be investigated at the same time, would facilitate understanding of the inflammatory process involved in both diabetes and periodontal diseases.

The aim of this study was to investigate the effect of type 2 diabetes on the local expression of inflammatory molecules involved in periodontal inflammation and healing by comparing GCF levels of 27 inflammatory molecules in patients with type 2 diabetes, with and without chronic periodontitis, and in chronic periodontitis subjects without diabetes. A putative correlation between HbA1c and the molecules under investigation was also explored. We tested the hypothesis that type 2 diabetes adversely influences the local expression of the inflammatory molecules under investigation.

## Methods

### Study design and participants

In all, 108 individuals were enrolled in the study, representing a randomly selected subset from 461 participants recruited for a previous study by Mohamed et al. [18]. The subjects were stratified into three groups: 54 with type 2 diabetes and chronic periodontitis (DM + CP), 30 with chronic periodontitis (CP) and 24 with type 2 diabetes (DM). The study participants were aged 24-70 years. Diabetes patients were recruited from The Jaber Abol’ez Diabetes Center in Khartoum-Sudan. Diabetes was diagnosed by specialist physicians at the center according to the criteria of The American Diabetes Association [19]. Whole blood samples obtained from patients with diabetes were analysed for HbA1c by boronate affinity chromatography using a commercially available kit (LabonaCheck^™^ A1c analyzer) [20]. The CP group was recruited from the out-patient dental clinic at the Khartoum Dental Teaching Hospital. Recruitment of study participants and eligibility criteria for enrolment have been described earlier [18]. Briefly, criteria for enrolment were (i), being diagnosed with type 2 diabetes for more than one year and attending a specialized diabetes clinic -for patients with diabetes- (ii), having at least 10 remaining teeth (iii), no antibiotic, no steroidal and/or non-steroidal anti-inflammatory medication used during the last 3 weeks (iv), not treated with immunosuppressive chemotherapy, no current acute illness, no professional periodontal treatment received during the last 6 months and no ongoing pregnancy or lactation. Demographic data were obtained from the study participants by means of a structured questionnaire.

The study protocol was approved by The Ministry of Health in The Sudan and The Norwegian Research Ethics Committee at The University of Bergen (2012/1470/REK Vest). The study participants were enrolled between July and December 2012. Written informed consent was obtained from each participant after the project objectives, the steps in the oral clinical examination and the sampling procedures had been explained. The participants were informed of their dental diagnosis and referred for appropriate dental treatment if indicated.

### Clinical periodontal examination

All clinical assessments, group allocations and sampling-site selection were undertaken by a single, calibrated examiner (HGM). The periodontal examination included all teeth except the 3^rd^ molars using a color-coded periodontal probe (N22, 2-4-6-8-10-12 mm markings), a color-coded Nabors furcation probe (NAB2, 3-6-9-12 mm markings), curette, mirror, probe, tweezers and cotton rolls. The clinical examination comprised dental plaque assessment using the Silness and Loe Index [21], bleeding on probing (BoP), scored as present or absent, and probing depth (measured from the gingival margin to the base of the periodontal pocket in millimeters) at four sites on each tooth (mesial, distal, buccal and lingual). Participants were diagnosed as having chronic periodontitis if they had at least two sites with bleeding pockets of ≥ 4 mm (not on the same tooth) [22, 23]. The oral examination was repeated for 20 participants randomly selected within 2 weeks. Intra-examiner reliability was assessed by Cohen's kappa coefficient [24]. Kappa value (κ) was 0.88 for chronic periodontitis (yes/no).

### GCF sampling

GCF samples were collected using perio-paper strips, (PERIOPAPER® Gingival Fluid Collection Strips, Oraflow Inc., New York, USA). Four samples, each representing a quadrant, were collected from each participant. The strip was inserted into the mesiobuccal site of the sulcus/pocket of the 1^st^ molar. If missing, the 2^nd^ molar, 2^nd^ premolar or 1^st^ premolar was sampled, respectively. Quadrants with no posterior teeth were excluded from the sampling. After the supra-gingival biofilm had been removed with sterile cotton pellets, the sites were dried and isolated with cotton rolls. The paper strips were inserted 2 mm into the sulcus/pocket and left in place for 30 s. Strips that were visually assessed as contaminated with blood or saliva were discarded. The 4 strips were immediately pooled in one tube, labeled and stored in liquid nitrogen for further analysis.

### Protein extraction and quantification

For protein extraction, Tween buffer (230 μl) was added to each of the tubes containing the 4 strips. The tubes were shaken for 30 min and then centrifuged for 10 min at 4 °C and 1400 rpm. The extracted protein was quantified using a commercially available kit and following the manufacturer's instructions (Pierce^™^ BCA Protein Assay Kit, Thermo scientific, Rockford, USA). Absorbance was measured at 560 nm on a plate reader (FLUOstar OPTIMA- BMG Labtech, Germany). Total protein per sample (4 strips) was calculated in micrograms (μg).

### Analysis and grouping of inflammatory molecules

Following protein extraction, GCF samples (20 μl each) were processed by multiplex immunoassay containing fluorescent dyed microspheres conjugated with monoclonal antibody specific for 27 inflammatory molecules (Bio-Plex Human Cytokine Assay; Bio-Rad Inc., Hercules, CA, USA) [25]. The following molecules were investigated: *IL-1β, IL-1ra, IL-2, IL-4, IL-5, IL-6, IL-7, IL-8, IL-9, IL-10, IL-12, IL-13, IL-15, IL-17, Eotaxin, Basic Fibroblast Growth Factor* (FGF)*, Granulocyte Colony Stimulating Factor* (G-CSF)*, Granulocyte-Monocyte Colony Stimulating Factor* (GM-CSF)*, Interferon-γ* (INF-γ)*, Interferon Inducible Protein-10* (IP-10)*, Monocyte Chemo-attractive Protein-1* (MCP-1)*, Macrophage Inflammatory Protein-1α* (MIP-1α)*, Macrophage Inflammatory Protein-1β* (MIP-1β)*, Platelet-Derived Growth Factor* (PDGF), *Regulated Upon Activation, Normally T-Expressed, and Presumably Secreted* (RANTES)*, Tumor Necrosis Factor-α* (TNF-α) and *Vascular Endothelial Growth Factor* (VEGF). Samples were diluted 1:4 (50 μl in total) and incubated with coupled beads. Complexes were washed, incubated with detection antibody and thereafter, with Streptavidin-Phycoerythrin. A range of 105876 – 0.29 pg/ml recombinant cytokines was used to establish the standard curves. The levels of the inflammatory molecules were measured on a multiplex array reader (Bio-Plex Workstation from Bio-Rad Laboratories). The final quantities were calculated using software provided by the manufacturer and were reported as picograms per 30 s (pg/30 s) [26].

Based on the biological effect of each molecule, the molecules under investigation were grouped as: pro-inflammatory cytokines (IL-1β, IL-6, IL-9, IL-12 and TNF-α), anti-inflammatory cytokines (IL-4 and IL-10), chemokines (IL-8, IP-10, MCP-1, MIP-1α, MIP-1β and RANTES) and T-helper 2/T-helper 1 ratio (Th-2/Th-1) (IL-4, IL-6, IL-9, IL-10/INF-γ, IL-2).

### Statistical analysis

Inter-group differences in demographic and clinical data were assessed using chi-square and Fisher’s exact test for categorical variables, one way analysis of variance (ANOVA) with post-hoc (Sidak) adjustment for multiple comparisons for normally distributed continuous variables, and Kruskal-Wallis and Mann-Whitney test for skewed data. Since the distribution of the levels of the studied molecules is skewed, the natural logarithm links were calculated and used to detect the differences between the study groups, and one way analysis of variance (ANOVA) with post-hoc (Sidak) was conducted. Generalized linear models (GLM) with Gaussian family and log function were used to adjust for the potentially confounding effect of age, gender, smoking status, dental plaque, BoP and total protein on the outcome (molecule quantities). A possible correlation between HbA1c and the inflammatory status was investigated using Pearson correlation. Stata 13 (StataCorp. 2013. Stata Statistical Software: Release 13. College Station, TX: StataCorp LP.) was used for data analysis. P values less than 0.05 were considered statistically significant.

## Results

The demographic characteristics and clinical parameters of the study groups are presented in Table 1. BoP was the only clinical parameter that differed significantly between the groups. It was higher in the DM + CP group than in the CP and DM groups. The following inflammatory molecules, which were not detected in more than 30 % of the GCF samples, were excluded from the analysis: (IL-1ra, IL-5, IL-13, IL-15 and eotaxin). The unadjusted means of the quantities of the detected molecules across the study groups are presented in Table 2. After adjustment for potential confounders (age, gender, smoking status, BoP, dental plaque index and total protein), the DM + CP group had higher levels of IL-8 and MIP-1β, and lower levels of TNF-α, IL-4, INF-γ, RANTES and IL-7 compared to the CP group. Moreover, the DM + CP group had lower levels of IL-6, IL-7 and G-CSF than the DM group. The DM group had higher levels of IL-10, VEGF, and G-CSF than the CP group. Both diabetes groups (DM + CP and DM) had lower levels of MIP-1α and FGF compared to chronic periodontitis subjects without diabetes (CP) (Table 3).

**Table 1:** Distribution of socio-demographic and clinical indicators by study groups

Variable	DM + CP (*n* = 54)	CP (*n* = 30)	DM (*n* = 24)
Age, mean (SE)^1^	54.76 (1.37)	55.37 (1.83)	50.79 (2.05)
Gender, % (n)^2^			
Male	42.59 (23)	60.00 (18)	29.17 (7)
Female	57.41 (31)	40.00 (12)	70.83 (17)
Education, % (n)^2^			
Illiterate	29.63 (16)	33.33 (10)	20.83 (5)
Literate	70.37 (38)	66.67 (20)	79.17 (19)
Employment, % (n)^2^			
Unemployed	62.96 (34)	43.33 (13)	70.83 (17)
Employed	37.04 (20)	56.67 (17)	29.17 (7)
Smoking, % (n)^3^			
Yes	12.96 (7)	26.67 (8)	12.50 (3)
No	87.04 (47)	73.33 (22)	87.50 (21)
Hypertension, % (n)^2^			
Yes	31.48 (17)	20.00 (6)	29.17 (7)
No	68.52 (37)	80.00 (24)	70.83 (17)
Regular dental attendance, % (n)^3^			
Yes	3.70 (2)	10.00 (3)	8.33 (2)
No	96.30 (52)	90.00 (27)	91.67 (22)
Duration of diabetes, mean (SE)^4^	8.44 (0.83)	-----	9.67 (1.70)
HbA1c %, mean (SE)^5^	9.17 (0.24)	-----	9.25 (0.49)
Plaque index, mean (SE)^1^	1.66 (0.05)	1.40 (0.06)	1.47 (0.07)
Percentage of teeth with BoP, mean (SE)^1^	58.88 (2.85)a	28.97 (3.82)b**	32.51 (4.28)b**
Pocket depth, % (n)^2^			
4-5 mm	59.30 (32)	70.00 (21)	0.00 (0)
≥6 mm	40.70 (22)	30.00 (9)	0.00 (0)
Pocket depth, mean (SE)^4^	4.16 (0.09)	4.25 (0.12)	-----
Total protein -μg, mean (SE)^6^	82.78 (7.53)	84.23 (15.25)	77.84 (9.06)

Different letters indicate statistically significant differences

^1^one-way ANOVA

^2^chi-square test

^3^Fisher’s exact test

^4^Mann-Whitney *U* test

^5^Independent sample *T* test

^6^Kruskal-Wallis test

** *P* < 0.01

**Table 2:** Levels of the detected inflammatory molecules among the study groups (pg/30s)

Inflammatory molecule, mean (SE)	DM + CP (*n* = 54)	CP (*n* = 30)	DM (*n* = 24)
IL-1β	350.50 (31.18)	260.31 (41.45)	261.74 (46.34)
IL-6	9.01 (0.83)a	11.12 (1.11)ab	12.67 (1.27)b*
IL-9	6.91 (0.57)	9.64 (0.76)	8.57 (0.87)
IL-12	13.93 (1.07)	16.44 (1.43)	17.13 (1.63)
TNF-α	17.70 (1.84)a	31.57 (2.47)b**	22.05 (2.82)ab
IL-4	0.83 (0.06)a	1.26 (0.09)b**	0.92 (0.10)ab
IL-10	27.74 (1.43)ab	23.68 (1.90)a	33.02 (2.17)b**
IL-2	8.01 (0.98)a*	8.93 (1.30)a*	12.68 (1.48)b
INF-γ	29.55 (2.61)a**	47.50 (3.50)b	33.89 (3.92)a*
IL-8	633.87 (48.22)	501.96 (62.25)	507.65 (69.60)
IP-10	19.32 (2.30)a	24.60 (3.01)ab	29.13 (3.43)b*
MCP-1	6.10 (0.87)a	10.73 (1.11)b**	7.88 (1.24)ab
MIP-1α	3.14 (0.27)a*	4.52 (0.36)b	2.98 (0.40)a*
MIP-1β	23.53 (2.64)	21.37 (3.54)	26.03 (3.95)
RANTES	32.93 (2.77)	45.64 (3.72)	40.12 (4.16)
FGF	76.91 (4.62)a**	105.16 (6.20)b	76.34 (6.93)a*
PDGF	13.05 (0.93)a	17.22 (1.24)b*	15.45 (1.39)ab
VEGF	212.01 (11.75)	188.50 (15.77)	222.83 (17.63)
IL-7	2.63 (0.31)a	4.85 (0.42)b**	4.24 (0.47)b**
G-CSF	122.73 (12.48)a	131.02 (16.75)ab	196.73 (19.13)b**
GM-CSF	621.28 (21.82)	558.27 (29.28)	555.30 (33.44)
IL-17	72.04 (3.71)a	246.03 (62.90)b**	79.98 (6.68)ab

Different letters indicate statistically significant differences using one-way ANOVA for natural log. links of the amounts of inflammatory molecules

*P < 0.05

**P < 0.01

**Table 3:** Adjusted means of the levels of inflammatory molecules (pg/30s)

Inflammatory molecule, mean (SE)	DM + CP (*n* = 54)	CP (*n* = 30)	DM (*n* = 24)
IL-6	9.01 (0.94)a	11.11 (1.30)ab	14.01 (1.50)b*
TNF-α	17.68 (2.06)a	31.64 (3.16)b**	23.13 (3.20)ab
IL-4	0.84 (0.07)a	1.25 (0.11)b*	0.96 (0.11)ab
IL-10	28.82 (1.66)ab	22.46 (1.99)a	33.72 (2.42)b**
INF-γ	30.62 (2.99)a	45.77 (4.29)b*	36.88 (4.62)ab
IL-8	699.48 (53.44)a	464.17 (57.03)b*	503.04 (71.04)ab
MIP-1α	3.05 (0.32)a*	4.79 (0.47)b	2.99 (0.45)a*
MIP-1β	28.71 (3.05)a	16.84 (2.86)b*	22.37 (3.40)ab
RANTES	31.94 (3.14)a	48.01 (4.70)b*	41.16 (4.79)ab
FGF	71.66 (5.17)a**	113.28 (7.90)b	80.86 (7.93)a**
VEGF	215.04 (12.69)ab	177.52 (16.40)a	255.82 (19.39)b**
IL-7	2.81 (0.35)a	4.47 (0.51)b*	4.78 (0.56)b*
G-CSF	131.71 (14.41)a*	116.98 (17.79)a**	206.60 (21.25)b

Different letters indicate statistically significant differences using GLM with Gaussian family and log. function adjusting for age, gender, smoking status, BoP, dental plaque index and total protein

*P < 0.05

**P < 0.01

**Fig. 1:**
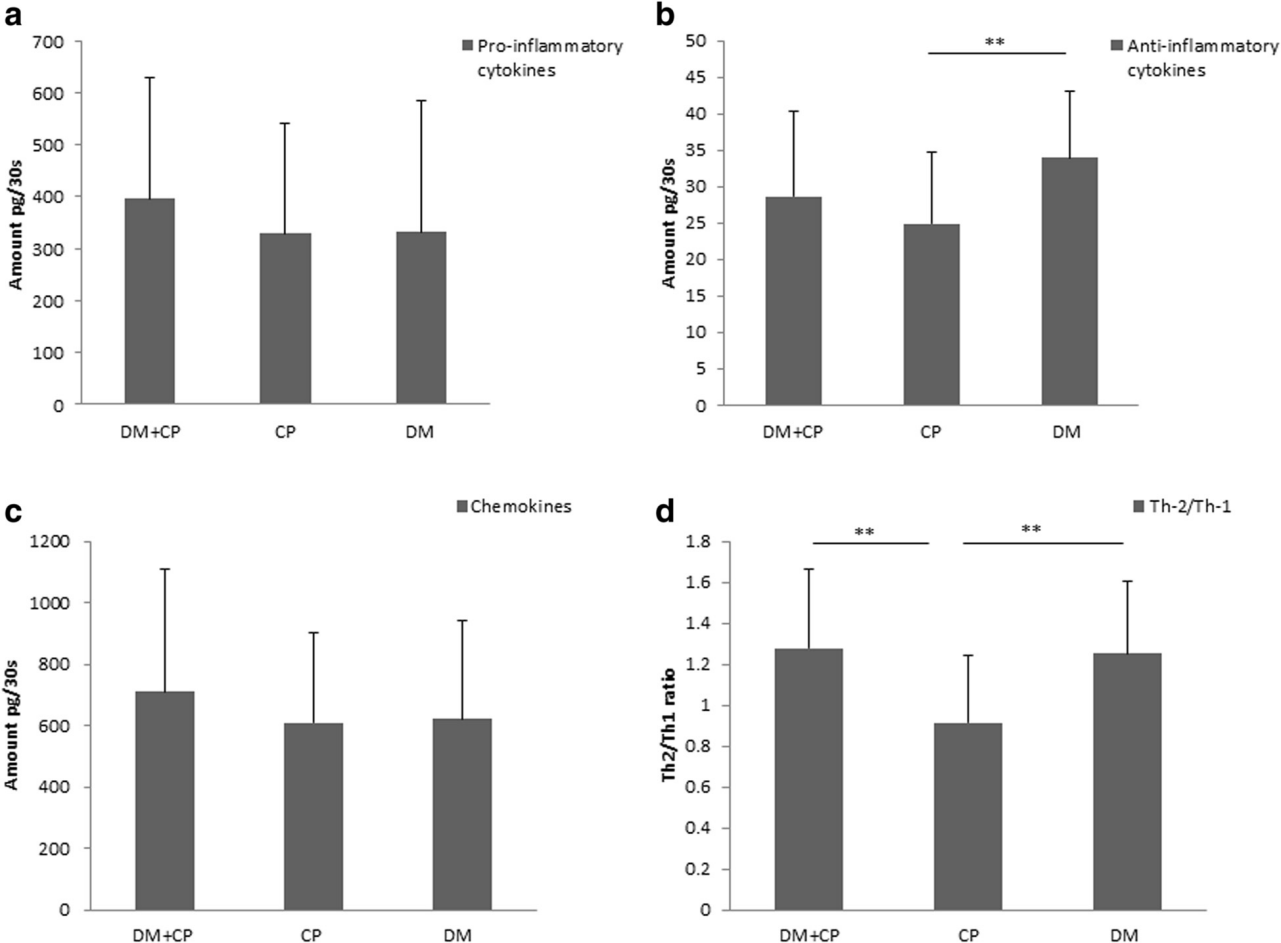
Levels of inflammatory molecules in GCF of the study groups. **a**: pro-inflammatory cytokines (IL-1β, IL-6, IL-9, IL-12, TNF-α), **b**: anti-inflammatory cytokines (IL-4, IL-10), **c**: chemokines (IL-8, IP-10, MCP-1, MIP-1α, MIP-1β, RANTES),** d:** Th-2/Th-1 ratio: (IL-4, IL-6, IL-9, IL-10) / (INF-γ, IL-2), **: *P* < 0.01

**Fig. 2:**
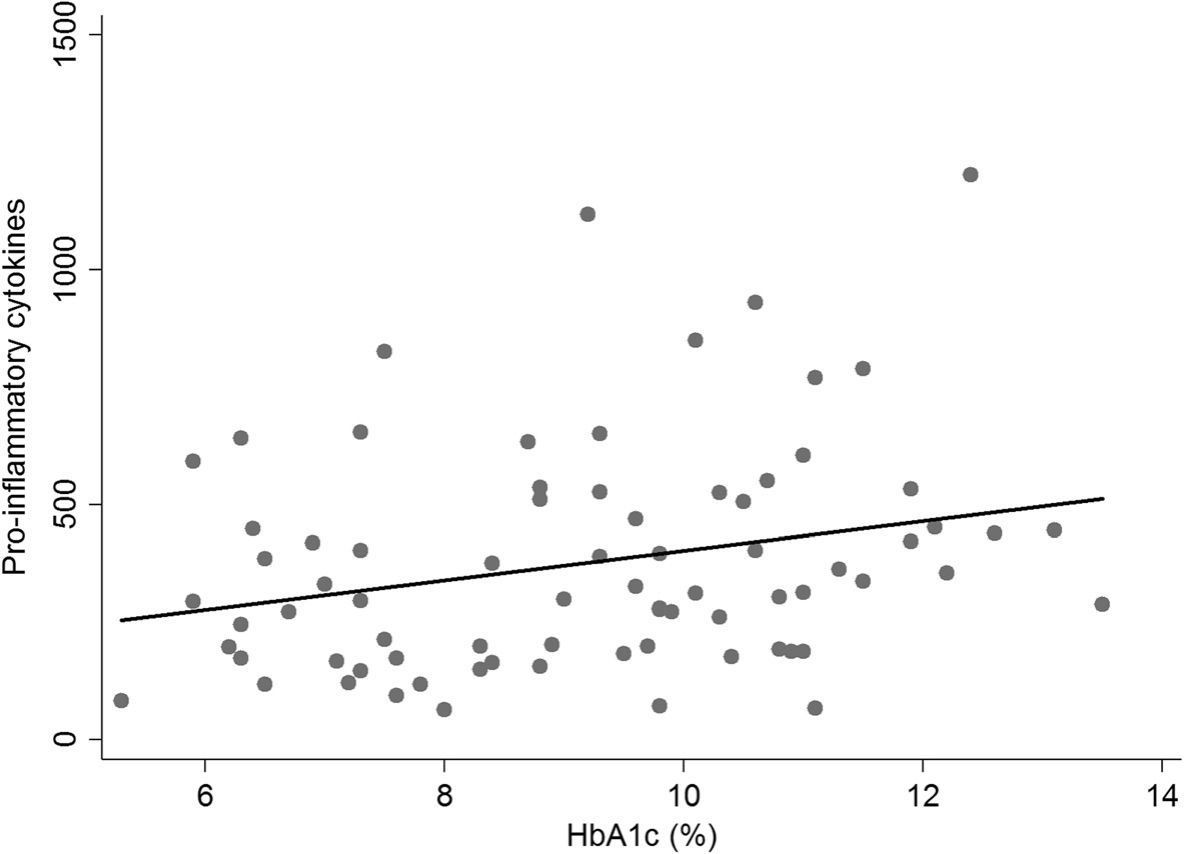
Correlation between HbA1c and the pro-inflammatory cytokines. Pearson correlation coefficient: 0.27, P value: 0.02

**Fig. 3:**
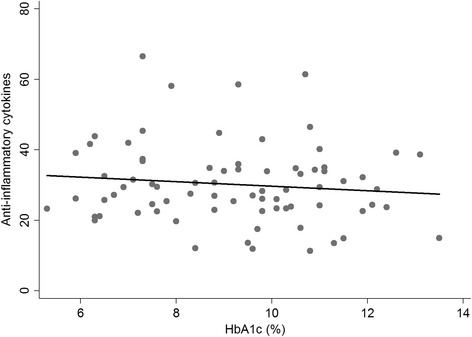
Correlation between HbA1c and the anti-inflammatory cytokines. Pearson correlation coefficient: -0.11, P value: 0.33

The Th-2/Th-1 ratio was significantly higher in the diabetes groups (DM + CP and DM) than in the CP group (Fig. 1d). A weak positive correlation was observed between HbA1c and the levels of the pro-inflammatory cytokines (Pearson correlation coefficient: 0.27, P value: 0.02) (Fig. 2), while the correlation between HbA1c and the anti-inflammatory cytokines was not statistically significant (Pearson correlation coefficient: -0.11, P value: 0.33) (Fig. 3).

## Discussion

Pro-inflammatory cytokines induce inflammatory response to various stimuli such as bacterial lipopolysaccharides [27]. In patients with diabetes, IL-1β is regarded as one of the key cytokines in inflammatory periodontal tissue destruction [28]. In the present study, the level of IL-1β was highest in the DM + CP group, albeit not statistically significant. A recent meta-analysis reported that type 2 diabetes patients with chronic periodontitis were found to have significantly higher GCF levels of IL-1β than their systemically healthy counterparts [29]. In contrast, others have failed to confirm an association between levels of IL-1β and diabetes in individuals with periodontal disease [30, 31]. These inconsistencies might be attributed to the difference in HbA1c levels and the duration of diabetes. Contradictory results were reported from studies of TNF-α levels in oral fluids among patients with diabetes and chronic periodontitis [12]. In the present study, the levels of both TNF-α and IL-7 were lower in the DM + CP group than in the CP group. In contrast, other studies have reported higher GCF levels of TNF-α and IL-7 in patients with type 2 diabetes than in systemically healthy individuals [29, 32]. In this context, it is of interest to note that the levels of pro-inflammatory cytokines and chemokines in the DM group were comparable with those in the CP group, reflecting a local active inflammatory process in the DM group (Fig. 1a–1c). A weak positive correlation was observed between HbA1c and the levels of pro-inflammatory cytokines. Another study reported a significant positive correlation between IL-1β levels in GCF and HbA1c [33].

Studying the effect of IL-6 and IL-10, particularly in cross-sectional studies, is complicated by the fact that both are multifunctional cytokines (i.e. pro- and anti-inflammatory) [34, 35]. IL-6 is involved in activation of osteoclasts and Th-17 cells [36]. In contrast, it also induces the production of IL-1ra, thus contributes to the anti-inflammatory process [37]. There is no evidence to support an association between increased levels of IL-6 and destructive periodontal disease among individuals with hyperglycemia [38]. However, Javed et al., [39] reported that up-regulation of IL-6 together with IL-1α could be associated with diabetes related periodontal tissue destruction. In the present study, the levels of the anti-inflammatory cytokine IL-4 were lower in the DM + CP group than in the CP group. It has been reported that IL-4 is down-regulated in patients with type 2 diabetes [40]. In addition, in an in-vivo animal model for ligament healing, IL-4 was found to contribute to the proliferative phase of healing [41].

Chemokines are small peptides that recruit immune cells from the circulation to the tissues as needed [42]. Most investigations of the role of chemokines have focused on IL-8 [12]. In the present study, IL-8 was up-regulated in the DM + CP group compared to the CP group. The same trend has been observed in an investigation of serum levels of IL-8 [43]. MIP-1α is a potent chemokine that attracts macrophages, T-cytotoxic and natural killer cells [44]. It was down-regulated in both diabetes groups (DM + CP and DM) compared to the CP group. In contrast, Duarte et al., [32] reported higher levels of MIP-1α in patients with poorly controlled type 2 diabetes compared to systemically healthy controls. The present study demonstrated lower levels of RANTES in the DM + CP group compared to the CP group. It was reported that levels of RANTES negatively correlate with increased inflammation [45].

Type 2 diabetes might adversely affect periodontal health by down-regulating molecules involved in periodontal tissue regeneration and healing process such as FGF and PDGF [46, 47]. FGF was detected in lower amounts in the diabetes groups (DM + CP and DM) than in the CP group. Moreover, the levels of VEGF were higher in the DM group compared to the CP group. This observation might be explained by the fact that oxidative stress induces the VEGF signaling in patients with type 2 diabetes [48]. Moreover, our VEGF results corroborate with a previous study which reported a non-significant trend towards increased VEGF in GCF of patients with type 2 diabetes and chronic periodontitis compared to systemically healthy individuals with chronic periodontitis [49]. In contrast, Guneri et al., [50] concluded that GCF levels of VEGF were elevated in subjects with chronic periodontitis regardless of their diabetic status.

According to the cytokines they produce, Th-cells are divided into Th-1, Th-2, Th-17 and T-regulatory cells. Th-1 secretes IL-2 and INF-γ, while Th-2 secretes IL-4, IL-6, IL-9, IL-10 and IL-13 [51, 52]. Only limited information is available about the role of Th-cells in type 2 diabetes patients with periodontitis [12]. Th-2/Th-1 ratio was higher in both diabetes groups (DM + CP and DM) than in the CP group, indicating enhanced humoral response and progression of periodontal disease among patients with type 2 diabetes via Th-2/B-cell axis [53, 54].

In the present study, the low levels of some of the pro-inflammatory molecules detected in type 2 diabetes patients compared to those without diabetes can be explained by the fact that some of the patients with diabetes were receiving insulin therapy, which might affect the local expression of inflammatory molecules [43, 55]. Moreover, the more pronounced clinical signs of periodontal disease in individuals with type 2 diabetes can be attributed to the disturbance in the balance between the molecules involved in active inflammatory process on the one hand, and the molecules involved in controlling inflammation, healing and regeneration of periodontal tissues on the other hand [56, 57]. Up to date, there is no ideal biomarker that can be nominated for disease detection or progression. Therefore, the interest has been shifted towards considering combinations of various host responses [11, 58]. In the present study, the GCF volume was not measured. Therefore, total GCF protein was used as a surrogate measure of the GCF volume in the multivariate analysis to control for the potential effect of variability of GCF volume on our results [59]. In addition, pocket depth and bleeding on probing were both used to define cases with periodontitis, as clinical attachment loss data were not available. Consequently, the effect of type 2 diabetes on the study outcome might be underestimated [60]. Nevertheless, it was reported that both periodontal pocket depth and BoP reflect the current disease status and are strongly related to the local inflammatory activity compared to clinical attachment loss, which reflects past disease experience [61–63].

## Conclusions

Type 2 diabetes and chronic periodontitis may adversely influence the GCF levels of inflammatory molecules synergistically as well as independently. Moreover, type 2 diabetes was associated with high Th-2/Th-1 ratio, and adversely influenced the local expression of molecules involved in the anti-inflammatory and healing processes. Further prospective studies are warranted to produce sufficient evidence to support the application of specific GCF biomarkers for prediction and prognosis of periodontal disease among patients with diabetes.

## Abbreviations

GCF: Gingival crevicular fluid
HbA1c: Glycated haemoglobin
AGE: Advanced glycation end products
BoP: Bleeding on probing
IL: Interleukin
FGF: Basic fibroblast growth factor
G-CSF: Granulocyte colony stimulating factor
GM-CSF: Granulocyte-monocyte colony stimulating factor
INF-γ: Interferon-γ
IP-10: Interferon inducible protein-10
MCP-1: Monocyte chemo-attractive protein-1
MIP: Macrophage inflammatory protein
PDGF: Platelet-derived growth factor
RANTES: Regulated upon activation, normally T-expressed, and presumably secreted
TNF-α: Tumor necrosis factor-α
VEGF: Vascular endothelial growth factor
Th: T-helper
ANOVA: Analysis of variance
GLM: Generalized linear model
